# *Bacillus subtilis* HU58 and *Bacillus coagulans* SC208 Probiotics Reduced the Effects of Antibiotic-Induced Gut Microbiome Dysbiosis in an M-SHIME^®^ Model

**DOI:** 10.3390/microorganisms8071028

**Published:** 2020-07-11

**Authors:** Massimo Marzorati, Pieter Van den Abbeele, Sarah S. Bubeck, Thomas Bayne, Kiran Krishnan, Aicacia Young, Dilip Mehta, Anselm DeSouza

**Affiliations:** 1 Center for Microbial Ecology and Technology (CMET), Faculty of Bioscience Engineering, Ghent University, Coupure Links 653, 9000 Ghent, Belgium; Massimo.Marzorati@prodigest.eu; 2 ProDigest, Technologiepark 82, 9052 Zwijnaarde, Belgium; Pieter.VandenAbbeele@prodigest.eu; 3 Bubeck Scientific, 194 Rainbow Drive #9418, Livingston, TX 77399, USA; 4 Microbiome Labs, 101 E Town Pl, Saint Augustine, FL 92092, USA; tom@microbiomelabs.com (T.B.); kiran@microbiomelabs.com (K.K.); ayoung@microbiomelabs.com (A.Y.); 5 Synergia Life Sciences, PVT Ltd., Universal Majestic, 1503, PL Lokhande Marg, Chembur, Mumbai, Maharashtra 400071, India; DilipMehta39@hotmail.com (D.M.); Kay2seven@gmail.com (A.D.)

**Keywords:** gut microbiome, gut barrier, gut inflammation, probiotics, immunomodulation, dysbiosis, M-SHIME

## Abstract

Benefits associated with probiotic use have been reported; however, the mechanisms behind these benefits are poorly understood. The effects of a probiotic formulation (MegaDuo™) containing *Bacillus coagulans* SC208 and *Bacillus subtilis* HU58 on intestinal permeability and immune markers was assessed using a combination of the in vitro gut model, the mucosal simulator of the human intestinal microbial ecosystem (M-SHIME^®^), and an in vitro inflammatory bowel disease-like Caco-2/THP1 co-culture model in both healthy and antibiotic-induced dysbiosis conditions. Established M-SHIME^®^ proximal colon vessels were treated with/without clindamycin (1 week) and then with/without daily MegaDuo™ treatment (2 weeks). The mucosal and luminal microbial communities were sampled weekly. Suspensions were removed from the proximal colon vessels after 1 and 2 weeks of MegaDuo™ treatment and added to the co-culture system. Transepithelial resistance (membrane barrier function), cytokine/chemokine release, and NFκB activity were then measured. Under conditions of antibiotic-induced dysbiosis, suspensions from MegaDuo™ treated vessels showed reduced gut membrane barrier damage and decreased levels of TNFα and IL-6 compared with suspensions from untreated vessels; no appreciable differences were observed under healthy conditions. MegaDuo™ treatment had no effect on NFκB activity of THP1-Blue™ cells. The potential benefits of MegaDuo™ treatment appeared most evident after 2 weeks of treatment.

## 1. Introduction

The gut microbiome is a biologically active community that is important for maintaining intestinal homeostasis. Most of the organisms making up the gut microbiome reside in the colon [1] and they play important roles in nutrient and drug metabolism, immunomodulation, maintenance of gut barrier integrity, and protection from pathogenic bacteria [2]. A healthy gut microbiome should be highly diverse, stable, resistant to stress-related changes, and have a high level of redundancy for metabolic pathways [3,4].

The gut barrier is comprised of immune cells, intestinal bacteria, and epithelial cells held together by intercellular tight junctions. It protects against toxins and pathogenic bacterial and is involved in immunomodulation [2]. When intact, this barrier controls trafficking of molecules between the lumen of the gut and the lamina propria.

Short chain fatty acids (SCFAs), including acetate, propionate, and butyrate, are generated by bacterial fermentation in the colon where they exert beneficial effects on the intestinal epithelial barrier integrity and reduce intestinal inflammation [5]. In vitro, butyrate was shown to increase the transepithelial electrical resistance (TEER) of Caco-2 monolayers in a concentration-dependent manner [6]. Pro-inflammatory cytokines, like tumor necrosis factor (TNF)α and interleukin-1β (IL-1β), significantly contribute to the inflammation observed in inflammatory bowel diseases (IBD) [7]. Furthermore, these pro-inflammatory cytokines induce the production of chemokines, like chemokine (C–X–C motif) ligand (CXCL)-10, IL-8, and monocyte chemoattractant protein 1 (MCP1), which are involved in neutrophil recruitment and reactive oxygen species production. Finally, a decrease in anti-inflammatory signaling also contributes to the development of IBD [8]. Indeed, IL-10 is a critical anti-inflammatory cytokine involved in maintaining intestinal immune homeostasis. IL-10 exerts pleiotropic immunosuppressive effects, as it inhibits the release of pro-inflammatory cytokines by macrophages, antigen presentation, and T cell differentiation. Furthermore, IL-10 promotes regulatory T cell differentiation. Intestinal inflammation has a significant impact on barrier integrity, and inflammation-induced barrier disruption can be mimicked in vitro by using a Caco-2/THP1 co-culture model [9]. In this model, anti-TNFα antibodies inhibited the THP1-induced barrier disruption, indicating the crucial role of TNFα in barrier dysfunction. Furthermore, butyrate decreased lipopolysaccharide (LPS)-induced TNFα and IL-1β production in THP1 cells, whereas it increased the LPS-induced IL-10 production and increased the LPS-induced NFκB activation in these cells [10]. Additionally, butyrate significantly increased toll-like receptor (TLR4)-mediated NFκB signaling in NCI-h716 cells by increasing the expression of the TLR4 receptor [11]. Finally, butyrate was shown to decrease pro-inflammatory cytokine and chemokine expression in several immune cell types [12].

Disruptions to a healthy gut microbiome can result in dysbiosis (gut microbiome imbalance), which is associated with many common diseases including IBD, irritable bowel syndrome, non-alcoholic fatty liver disease, and metabolic diseases such as obesity [13]. Exposure to antibiotics can cause dysbiosis of the gut microbiome, which results in a decrease in taxonomic diversity and changes in the numbers of individual microorganisms [14]. This imbalance can induce intestinal inflammatory responses, which may become pathological [15]. Antibiotics may also cause changes in the function of the gut barrier [15,16]. Such changes include translocation of gut bacteria across the colonic epithelium, which further contributes to increased inflammation [15].

Probiotics are defined as “live microorganisms which when administered in adequate amounts confer a health benefit to the host” [17]. They can help improve gastrointestinal health, improve gut barrier function, and affect both the mucosal and systemic immune systems [18]. Certain *Bacillus* strains have recently been recognized both as part of the normal flora and as having probiotic effects [19,20]. *Bacillus* spp. have several characteristics that make them suitable for probiotic use including a high level of resistance to desiccation and heat, as well as stability over a wide range of pHs [19]. Multiple health benefits have been attributed to the use of *Bacillus* spp. as probiotics, though the mechanisms of these benefits are poorly understood. Clinical trials evaluating the probiotic use of *Bacillus* spp. have been reviewed by Elshaghabee et al. [20].

*Bacillus coagulans* was first isolated from spoiled milk [21] and exhibits characteristics of both the *Bacillus* and the *Lactobacillus* genera [22]. It is a Gram-positive, rod shaped, spore-forming, facultative anaerobic, lactic-acid producing bacteria [22,23]. The probiotic activity observed for this species is likely due to lactic acid and other enzymes produced by the bacterium. *B. coagulans* has been reported to be safe by multiple regulatory agencies including the US Food and Drug Administration [24].

*Bacillus subtilis* has been isolated both from the soil and the intestinal tracts of humans [25,26,27]. It is a Gram-positive, rod shaped, spore-forming aerobic/facultative anaerobic bacterium [25]. *B. coagulans* and *B. subtilis* can be found either alone or in combination with other probiotics in multiple commercially available formulations [20]. It is thought that probiotics containing a combination of two or more spore-forming *Bacillus* species may improve the effects of treatment versus those containing a single strain. A recent study reported that when patients with dietary endotoxemia took a probiotic supplement containing five spore-forming species of *Bacillus* (*Bacillus indicus* HU36, *B. subtilis* HU58, *B. coagulans*, *Bacillus licheniformis*, and *Bacillus clausii*) for 30 days, they experienced a significant reduction in intestinal permeability as evidenced by significant reductions in endotoxins, triglycerides, and proinflammatory cytokines [28].

To support the use of probiotics in human health, it is important to understand specifically what benefits they impart. The aim of the present study was to assess the effects of MegaDuo™, a probiotic formulation containing *B. coagulans* SC208 and *B. subtilis* HU58, on intestinal permeability and immune markers using an in vitro gut model, the mucosal simulator of the human intestinal microbial ecosystem (M-SHIME^®^) [29], in combination with an in vitro IBD-like Caco-2/THP1 co-culture model [9] under healthy gut conditions or following antibiotic-induced dysbiosis.

## 2. Materials and Methods

### 2.1. M-SHIME^®^ Model.

The M-SHIME^®^ reactor configuration was adapted from the SHIME^®^ model (ProDigest, Ghent, Belgium and Ghent University, Ghent, Belgium) previously described by Molly et al. [30] to include modelling of both the mucus-associated and luminal microbial communities [29,31]. Details of inoculum preparation, retention times, pH, temperature settings, mucin-covered microcosms, and reactor feed composition have been previously described [29,32]. The present study included minor modifications to Van den Abbeele et al. [29], which are described as follows. Briefly, the reactor consisted of three double-jacketed vessels that were connected through peristaltic pumps and operated completely anaerobically. These vessels simulate the stomach and small intestine, and for the purposes of our study, the colon vessels were limited to the proximal colon (pH 5.6–5.9, retention time 20 h). To simulate the gut microbiome, the proximal colon vessels were inoculated with microbiota isolated from an appropriate fecal sample originating from a single healthy adult volunteer with no history of antibiotic treatment in the 6 months prior to sample collection.

The first vessel was fed nutritional medium supplemented with 2 g/L L-alanine to allow optimal germination of *Bacillus* spores. During the simulation of the small intestine, the system was fed pancreatic and bile liquid. Feedings were conducted three times a day using a fill-and-draw method. After digestion in the upper gastrointestinal tract, the slurry was pumped into the proximal colon vessel. Proximal colon vessels were continuously stirred and monitored for constant volume and pH. All vessels were maintained at 37 °C.

**Figure 1:**
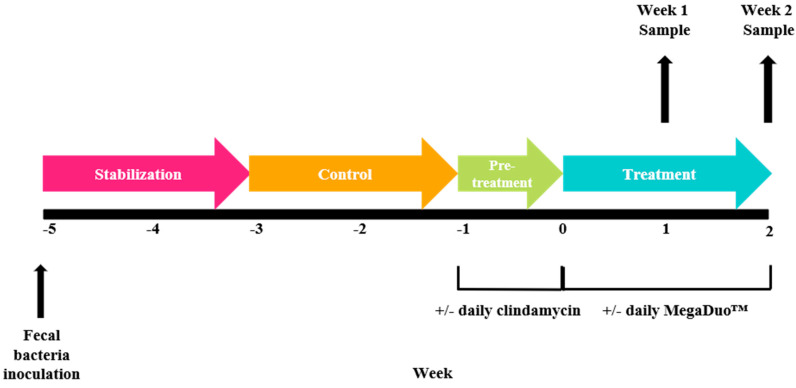
Schematic of the experimental design.

The experimental schedule is schematically shown in Figure 1. There was a 2-week stabilization period to allow the microbiota to adapt to the in vitro environment, followed by a 2-week control period during which stability in the microbiome was established and baseline parameters were measured. At the completion of the control period, there was a 1-week pre-treatment period. Clindamycin (33.9 mg/mL, thrice daily) was added to one proximal colon vessel for 7 days to induce dysbiosis of the microbiota, which is characteristic of many gastrointestinal tract diseases. The other proximal colon was operated under normal healthy conditions, without antibiotics. Next, the 2-week treatment period was initiated, and MegaDuo™ (combination of *B. coagulans* SC208 and *B. subtilis* HU58; powder dissolved in gastric fluid; Synergia Life Sciences, Mumbai, Maharashtra, India) was administered once daily (first feeding cycle, 2 × 10^9^ CFU/day) over the 2-week treatment period. Proximal colon samples were collected after 1 and 2 weeks of treatment. Supernatants were centrifuged (5 min, 9000 RPM) and stored at –20 °C.

### 2.2. Microbial Community Analysis

Once per week, from the start of the control period to the end of the treatment period, samples were collected for microbial community analysis from both the luminal and mucosal compartment of each vessel. DNA was isolated from 0.1 g of the mucosal compartment sample or pelleted cells originating from a 1 mL luminal sample using the method described by Vilchez-Vargas et al. [33]. Next, quantitative polymerase chain reaction (qPCR) for Bacteroides phylum, Firmicutes phylum, *Enterobacteriaceae* family, *Bifidobacterium* spp., and *Lactobacillus* spp. was performed using a StepOnePlus™ real-time PCR system (Applied Biosystems, Foster City, CA, USA). Each sample was analyzed in technical triplicate, and outliers (more than 1 CT difference) were omitted. qPCR was conducted according to previously described methods: Firmicutes and Bacteroidetes phyla, Guo et al. [34]; *Bifidobacterium* spp., Rintillä et al. [35]; *Lactobacillus* spp., Furet et al. [36]; *Enterobacteriaceae*, Nakano et al. [37].

Microbiota profiling of the colon compartments was accomplished using 16S-targeted Illumina sequencing analysis. The 16S rRNA gene V3–V4 hypervariable regions were amplified by PCR using primers 341F (5′-CCT ACG GGN GGC WGC AG-3′) and 785Rmod (5′-GAC TAC HVG GGT ATC TAA KCC-3′); to increase coverage, the reverse primer was adapted from Klindworth et al. [38]. Quality control PCR was performed using Taq DNA polymerase with the Fermentas PCR Kit according to the manufacturer’s instructions (Thermo Fisher Scientific, Waltham, MA, USA). Genomic DNA extracts (10 μL) were sent to LGC genomics GmbH (Germany) for library preparation and sequencing on an Illumina MiSeq platform with v3 chemistry with the above-mentioned primers [38].

Read assembly and cleanup for the 16S-targeted sequencing analysis was derived from the MiSeq protocol described by the Schloss lab [39,40]. In brief, mothur (v. 1.39.5) was used to assemble reads into contigs, perform alignment-based quality filtering (alignment to the mothur-reconstructed SILVA SEED alignment, v. 123), remove chimeras, assign taxonomy using a naive Bayesian classifier [41] and SILVA NR v128, and cluster contigs into operational taxonomic units (OTUs) at 97% sequence similarity. All sequences classified as Eukaryota, Archaea, chloroplasts, and mitochondria were removed, as well as sequences that could not be classified. For each OTU, representative sequences were selected as the most abundant sequence within that OTU.

### 2.3. Caco-2/THP1-Blue™ Co-Culture Model.

Co-culture experiments using Caco-2 (HTB-37, American Type Culture Collection, Manassas, VA, USA) and THP1-Blue™ cells (InvivoGen, San Diego, CA, USA) were performed as previously described [42]. Briefly, a semi-permeable insert (pore size 0.4 µM) with a monolayer of Caco-2 cells (TEER >300 Ω cm^2^ (epithelial volt-ohm meter, Millipore, Burlington, MA, USA)) was placed into a well of phorbol 12-myristate 13-acetic acid (PMA)-differentiated THP1-Blue™ cells (100 ng/mL, 48 h). The apical compartment (containing Caco-2 cells) was filled with sterile-filtered (0.22 µM) colonic M-SHIME^®^ suspensions (diluted 1:5 *v/v* in Caco-2 complete medium) or treated with 12 mM sodium butyrate (NaB) (Sigma-Aldrich, St. Louis, MO, USA) for a positive control and complete Caco-2 medium for a negative control. The basolateral compartment (containing THP1-Blue™ cells) was filled with complete Caco-2 medium. Co-cultures were then placed in a humidified incubator at 37 °C, 5% CO^2^. Twenty-four hours later, the TEER was measured for each well. The percent of the initial value (0 h) for each well was calculated using the following formula:

(24 h Ω cm^2^ − empty insert Ω cm^2^/0 h Ω cm^2^) × 100

The basolateral media was then discarded and replaced with Caco-2 complete medium containing 500 ng/mL ultrapure LPS (*Escherichia coli* K12, InvivoGen, San Diego, CA, USA) to stimulate the cells. Medium containing 500 ng/mL LPS and 1 µM hydrocortisone (HC, Sigma-Aldrich, St. Louis, MO, USA) or medium without LPS were used as controls. After 6 h, the basolateral supernatants were collected and processed for the measurement of cytokines, chemokines, and NFκB. Co-culture experiments were performed in technical triplicates

### 2.4. Measurement of NFκB Activity

THP1-Blue™ cells are stably transfected with an NFκB reporter construct in which the secreted alkaline phosphatase (SEAP) gene is under the control of an NFκB inducible promoter. When NFκB is activated, expression of SEAP is induced. SEAP was measured in co-culture basolateral supernatants using the QUANTI-Blue reagent (InvivoGen, San Diego, CA, USA) according to the manufacturer’s instructions.

### 2.5. Cytokine and Chemokine Measurements

Levels of human IL-1β, IL-8, IL-10, TNFα, CXCL10, and MCP1 in the co-culture basolateral supernatants were measured using Luminex^®^ multiplex (Affymetrix-eBioscience, Waltham, MA, USA). Assays were performed according to the manufacturer’s instructions.

### 2.6. Statistical Methods

For the TEER controls, complete Caco-2 media (negative) and NaB (positive) values were compared using an unpaired, two-tailed Student’s *t*-test. For the co-cultures treated with colonic M-SHIME^®^ suspensions, treatment samples were compared to the M-SHIME^®^ control (M-SHIME^®^ media) using an ordinary one-way analysis of variance (ANOVA) with Dunnet’s multiple comparisons test. Regarding the controls, cytokine levels and NFκB activity, LPS–, LPS + HC, and LPS + NaB were compared to LPS+; for the colonic M-SHIME^®^ suspensions, treatment samples were compared to the M-SHIME^®^ control (M-SHIME^®^ media) using an ordinary one-way ANOVA with Dunnett’s multiple comparisons test. A *p*-value < 0.05 was considered statistically significant; *, **, ***, and **** represent *p* < 0.05, *p* < 0.01, *p* < 0.001 and *p* < 0.0001, respectively. All statistics were performed using GraphPad Prism version 7.02 for Windows (GraphPad Software, San Diego, CA, USA).

## 3. Results

### 3.1. Microbial Community Analysis

**Figure 2:**
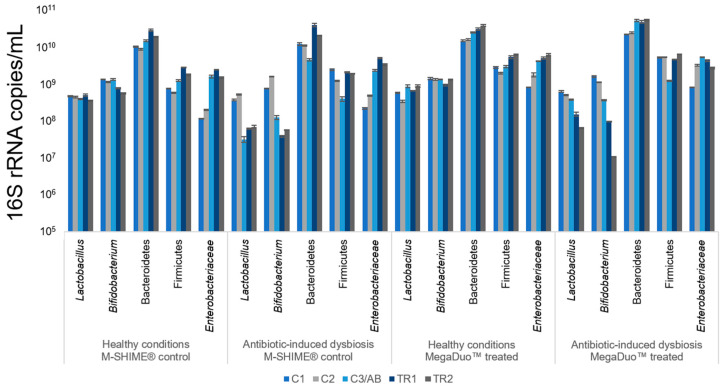
Luminal microbial community composition as assessed by qPCR. All data are shown as 16S rRNA copies/mL in the luminal phase of the proximal colon. All samples were analyzed in triplicate. Data are shown as mean ± standard deviation. C1 = control Week 1; C2 = control Week 2; C3/AB = control Week 3 (healthy condition, pre-treatment)/antibiotic (antibiotic-induced dysbiosis condition, pre-treatment); TR1 = treatment Week 1; TR2 = treatment Week 2; M-SHIME^®^ = mucosal simulator of the human intestinal microbial ecosystem.

The luminal microbiota composition for the duration of the study is shown in Figure 2. *Lactobacillus* and *Bifidobacterium* spp. remained constant throughout the study period under healthy conditions (no clindamycin). The addition of clindamycin decreased these levels, and they did not recover after cessation of antibiotic treatment, even with MegaDuo™ treatment. Under healthy conditions, Bacteroidetes and Firmicutes levels were increased upon treatment with MegaDuo™. No clear effects on the Bacteroidetes levels, and a decrease in Firmicutes levels occurred with clindamycin exposure. Firmicutes levels recovered in both the MegaDuo™ treated and M-SHIME^®^ control lumens. Under healthy conditions, *Enterobacteriaceae* levels gradually increased in both the MegaDuo™ treated and M-SHIME^®^ control lumens, and clindamycin exposure resulted in an increase in these levels. After cessation of clindamycin, MegaDuo™ treatment resulted in an immediate decrease of *Enterobacteriaceae*, indicating a faster recovery of these levels compared with the M-SHIME^®^ control. This is of importance as this family is most likely linked with enteric diseases.

**Figure 3:**
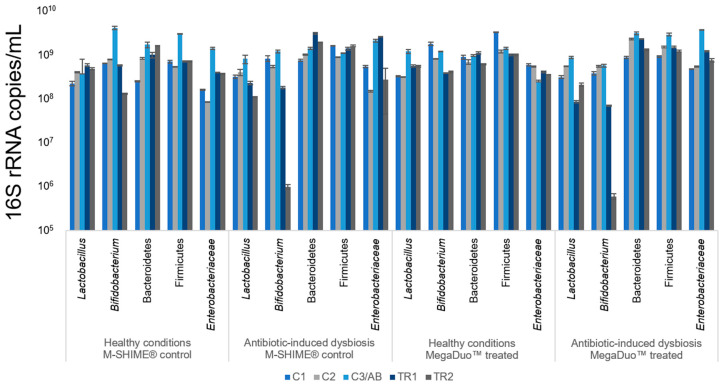
Mucosal microbial community composition as assessed by qPCR. All data are shown as 16S rRNA copies/mL in the mucosal phase of the proximal colon. All samples were analyzed in triplicate. Data are shown as mean ± standard deviation. C1 = control Week 1; C2 = control Week 2; C3/AB = control Week 3 (healthy condition, pre-treatment)/antibiotic (antibiotic-induced dysbiosis condition, pre-treatment); TR1 = treatment Week 1; TR2 = treatment Week 2; M-SHIME^®^ = mucosal simulator of the human intestinal microbial ecosystem.

The mucosal microbiota composition for the duration of the study is shown in Figure 3. Under healthy conditions, there were no notable differences in microbiota composition between MegaDuo™ treated and M-SHIME^®^ control chambers. After clindamycin exposure, most bacterial groups increased, indicating a protective effect of the mucosal environment in terms of antibiotic exposure. After cessation of antibiotic treatment, similar effects as reported for the luminal microbiota were observed with MegaDuo™ treatment.

**Figure 4:**
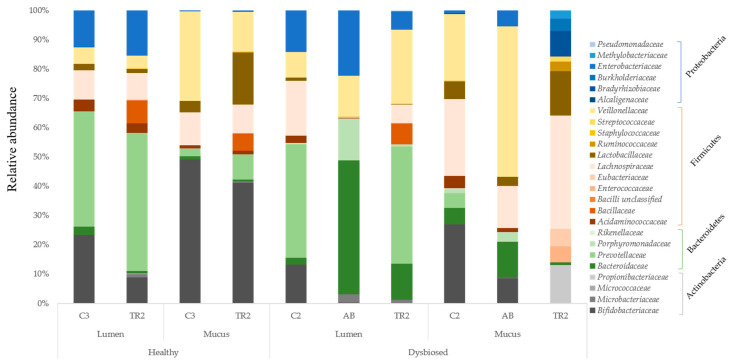
Relative abundance of different families belonging to specific phyla in the lumen and mucus of the proximal colon of the M-SHIME^®^ treated with MegaDuo™ under healthy and antibiotic-induced dysbiosed conditions. C3 = end of the control period (healthy condition); TR2 = end of the treatment period; C2 = end of the control period (dysbiosed condition); AB = end of the antibiotic period; M-SHIME^®^ = mucosal simulator of the human intestinal microbial ecosystem.

Microbial community composition at the family and OTU level is shown in Figure 4. Under healthy conditions, MegaDuo™ treatment resulted in a decrease in abundance of the *Bifidobacteriaceae* and the *Veillonellaceae* families and an increase in abundance of the *Prevotellaceae* and *Baccillaceae* families in both the luminal and mucosal environment. There was also an increase in the abundance of *Lactobacillaceae* in the mucus layer. Clindamycin exposure resulted in a decrease in Actinobacteria abundance and an increase in *Enterobacteriaceae* abundance; *Prevotellaceae* levels decreased below the detection limit in both the luminal and mucosal environments. There was also a drastic negative effect on the Firmicutes phylum. Treatment with MegaDuo™ under dysbiosed conditions resulted in an increase in bacterial families associated with propionate (SCFA) production, while *Enterobacteriaceae* levels decreased. There was an increase in the abundance of the *Bacillaceae* family in the lumen. Reciprocal Simpson Diversity Index data indicated that antibiotic treatment reduced the diversity of the gut microbial community, with the strongest effect in the mucosal compartment; MegaDuo™ increased this diversity, particularly following antibiotic treatment (Supplementary Materials Table S1).

### 3.2. Transepithelial Electrical Resistance

Caco-2/THP1-Blue™ co-cultures were exposed to controls or M-SHIME^®^ suspensions (+/− clindamycin pretreatment, 1 or 2 weeks of daily +/− MegaDuo™ dosing) for 24 h, and then THP1-induced damage to the Caco-2 membrane was measured. The controls (complete Caco-2 medium and NaB) performed as expected (Supplementary Materials Figure S1). NaB treatment protected the Caco-2 cells from THP1-induced damage, maintaining the barrier integrity of the monolayer; the TEER was significantly higher in the NaB treated co-cultures compared with the Caco-2 medium control (*p* < 0.0001).

**Figure 5:**
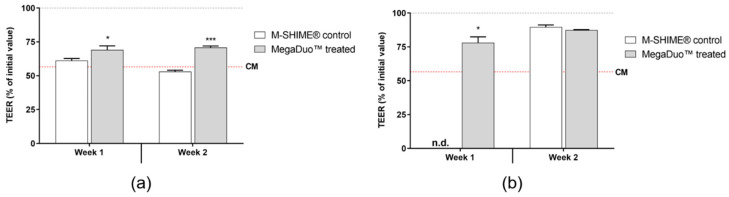
Barrier integrity of Caco-2 cells after exposure to M-SHIME^®^ suspensions (**a**) with or (**b**) without pre-treatment with clindamycin. * *p* ≤ 0.05, *** *p* ≤ 0.001, grey dashed line = 100% TEER, red dashed line = TEER (% of initial value) for co-cultures treated with CM. Error bars represent standard error of the mean. CM = Caco-2 media; M-SHIME^®^ = mucosal simulator of the human intestinal microbial ecosystem; TEER = transepithelial electric resistance.

Exposure of the co-cultures to +clindamycin suspensions without MegaDuo™ dosing resulted in THP1-induced damage to the Caco-2 membrane barrier at a level similar to that observed for the complete Caco-2 media control, while exposure to +clindamycin suspensions dosed with MegaDuo™ resulted in protection of the Caco-2 membrane integrity relative to both the M-SHIME^®^ and Caco-2 media controls (Figure 5a). The protection was significant whether the suspensions were collected 1 week (*p* = 0.0314) or 2 weeks (*p* = 0.0002) after MegaDuo™ dosing (MegaDuo™ vs. M-SHIME^®^ control). The effect was more pronounced at 2 weeks, with MegaDuo™ exposed co-cultures showing a Caco-2 membrane TEER value ~70% of the initial value (pre-exposure). The controls (M-SHIME^®^ control or Caco-2 media) had TEER values closer to 60%. The results demonstrated a protective effect of MegaDuo™ treated M-SHIME^®^ suspensions against THP1-induced damage to the Caco-2 membrane barrier under conditions of antibiotic-induced dysbiosis.

M-SHIME^®^ suspensions that were not pretreated with clindamycin, whether or not they were dosed with MegaDuo™, were largely able to prevent THP1-induced damage to the membrane barrier integrity. Co-cultures exposed to the M-SHIME^®^ suspensions had TEER values of 80–90% of pre-exposure values compared with 57% for the co-cultures treated with complete Caco-2 media (Figure 5b). This indicates that in conditions representing a healthy microbiome, the metabolites present in the M-SHIME^®^ suspensions were sufficient to protect membrane barrier integrity. Note that the control M-SHIME^®^ supernatants at Week 1 were suspected to have been contaminated during sample processing (i.e., after the collection of the samples) and were therefore not included in the analysis. The microbiota composition was similar for the control M-SHIME^®^ vessel at Week 1 and Week 2 (Figure 2 and Figure 3, TR1 vs. TR2), allowing for the use of Week 2 data as a comparator in place of Week 1 data.

### 3.3. Immune Markers

Caco-2/THP1-Blue™ co-cultures were exposed to controls or M-SHIME^®^ suspensions (+/− clindamycin pretreatment, after 1 or 2 weeks of daily +/− MegaDuo™ dosing) for 24 h, and then LPS was added to the cultures; 6 h later, supernatants were collected and assayed for cytokines and chemokines. The controls worked as expected. LPS significantly increased secretion of all cytokines and chemokines tested relative to the complete Caco-2 media control except IL-10, which was significantly reduced (LPS+ vs. LPS–: TNFα, *p* = 0.003; IL-8, *p* = 0.0194; MCP1, *p* = 0.0245; CXCL10, *p* = 0.0005; IL-10, *p* = 0.0054; IL-6, *p* < 0.0001). HC was able to significantly suppress LPS-induced cytokines and chemokines (LPS+ vs. LPS+HC: TNFα, *p* = 0.004; IL-8, *p* = 0.0069; MCP1, *p* = 0.0078; CXCL10, *p* = 0.0017; IL-10, *p* = 0.0076; IL-6, *p* < 0.0001). NaB had selective effects; it was able to significantly increase LPS-induced IL-10 (*p* < 0.0001) and IL-6 (*p* = 0.0124) and to significantly decrease LPS-induced TNFα (*p* = 0.003), IL-8 (*p* = 0.0421), MCP1 (*p* = 0.0011) and CXCL10 (*p* = 0.0004), which are involved in inflammation and recruitment of immune cells (Supplementary Materials Figures S2A–F).

The effects of M-SHIME^®^ suspensions on cytokine and chemokine release in the co-culture system are reported in Table 1 and Table 2. The levels of some LPS-induced cytokines and chemokines were differentially affected by MegaDuo™ and control M-SHIME^®^ suspensions that were pretreated with clindamycin. We observed no differences in the levels of TNFα, IL-8, or IL-10, a slight reduction in MCP1 and CXCL10, and a significant reduction in IL-6 (*p* = 0.007) for MegaDuo™ treated versus M-SHIME^®^ control suspensions collected after 1 week of daily MegaDuo™ dosing. After 2 weeks of daily dosing, we observed no differences in IL-8, MCP1, CXCL10, or IL-6, a slight increase in IL-10, and a significant reduction in TNFα (*p* = 0.0067) for MegaDuo™ treated versus M-SHIME^®^ control suspensions (Table 1).

In the absence of clindamycin pretreatment (representing healthy gut conditions), exposure of co-cultures to suspensions after MegaDuo™ treatment resulted in no change for CXCL10, IL-10, or IL-6 and a significant reduction in TNFα (*p* = 0.0047), IL-8 (*p* = 0.0238), and MCP1 (*p* = 0.0359) in M-SHIME^®^ suspensions after 1 week of treatment (compared with M-SHIME^®^ control suspensions collected at Week 2; data collected using M-SHIME^®^ control suspensions from Week 1 were suspected to have been contaminated during sample processing (i.e., after sample collection) and were therefore not used for data analysis). The microbiota composition was similar for the control M-SHIME vessel at Week 1 and Week 2 (Figure 2 and Figure 3, TR1 vs. TR2), allowing for the use of Week 2 data as a comparator in place of Week 1 data. There was no change in the levels of TNFα, IL-8, CXCL10, IL-10, and IL-6 and a significant increase in MCP1 (*p* = 0.0304) after 2 weeks of treatment compared with the M-SHIME^®^ control (Table 2).

The supernatants collected for the cytokine and chemokine assays were used to determine the NFκB activity of THP1-Blue™ cells. The controls worked as expected (Supplementary Materials Figure S3). LPS increased NFκB activity (LPS+ vs. LPS–; *p* = 0.0020), and HC was able to reduce LPS-induced activity (LPS+ vs. LPS+HC; *p* = 0.0271). NaB increased NFκB activity in the presence of LPS (LPS+ vs. LPS + NaB; *p* = 0.0003).

**Figure 6:**
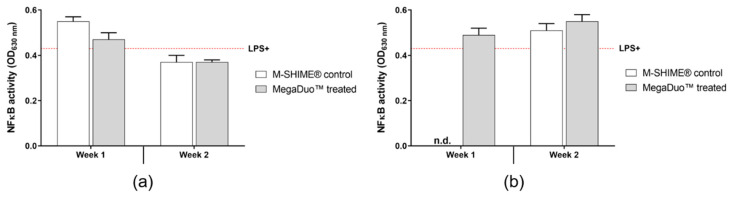
Effect of M-SHIME^®^ suspensions on NFκB activity of PMA-treated THP1 cells after LPS stimulation in the Caco-2/THP1-Blue™ co-culture model (**a**) with or (**b**) without pre-treatment with clindamycin. Red dashed line = LPS (no M-SHIME^®^ supernatants). Error bars represent standard error of the mean. LPS = lipopolysaccharide; M-SHIME^®^ = mucosal simulator of the human intestinal microbial ecosystem.

The effects of the M-SHIME^®^ suspensions on the NFκB activity of PMA-activated THP1 cells after 6 h LPS stimulation are shown in Figure 6. When pretreated with clindamycin, M-SHIME^®^ suspensions (M-SHIME^®^ control or MegaDuo™) collected after 1 week of MegaDuo™ dosing induced a slight increase in THP1 NFκB activity, and those collected after 2 weeks of dosing induced a slight decrease in activity compared to the LPS+ control (Figure 6a).

Suspensions not pretreated with clindamycin and collected after Week 1 and 2 of MegaDuo™ dosing, and M-SHIME^®^ control suspensions collected after Week 2 induced a slight increase in NFκB activity compared to the LPS+ control (Figure 6b). Note that the Week 1 M-SHIME^®^ control suspensions were suspected to have been contaminated during sample processing (i.e., after sample collection) and were therefore removed from analysis. The microbiota composition was similar for the control M-SHIME vessel at Week 1 and Week 2 (Figure 2 and Figure 3, TR1 vs. TR2), allowing for the use of Week 2 data as a comparator in place of Week 1 data.

There were no significant differences between the control and MegaDuo™ treated M-SHIME^®^ suspensions in terms of NFκB activity, indicating that any changes observed were related to metabolites present in both the control and treated M-SHIME^®^ suspensions and were independent of the MegaDuo™ treatment.

## 4. Discussion

We observed that a stable microbial community was established prior to clindamycin exposure and that clindamycin treatment induced changes to the microbial community that would be considered dysbiosis, including an increase in *Enterobacteriaceae*. MegaDuo™ treatment seemed to have a beneficial effect on recovery of the microbial community after antibiotic exposure, as evidenced by a more rapid decrease in *Enterobacteriaceae* and an increase in SCFA-producing Bacteroidetes and Firmicutes. MegaDuo™ treatment had beneficial effects on the microbial community under healthy conditions, which included increases in the abundance of Bacteroidetes and Firmicutes. There was an increase in the abundance of the *Bacillaceae* family in the lumen when MegaDuo™ treatment was given under conditions of dysbiosis and an increase in both the lumen and mucosal compartments after MegaDuo™ treatment under healthy conditions (vs. pre-treatment abundance). These results suggest that bacteria in the probiotic formulation may have grafted in the proximal colon chamber.

MegaDuo™ dosed M-SHIME^®^ suspensions provided an improvement in gut barrier integrity and reduced TNFα, MCP1, and IL-6 compared with control M-SHIME^®^ suspensions when under conditions of antibiotic-induced gut microbiome dysbiosis. We speculate that the reductions in cytokines may be attributed to SCFA production by Firmicutes, which recovered to a greater extent in the lumen after MegaDuo™ treatment compared with no treatment. No significant differences were observed in NFκB activity between MegaDuo™ treated and M-SHIME^®^ control suspensions, and no significant changes in IL-10 levels were observed regardless of the experimental conditions. In healthy gut conditions (without antibiotic treatment), the pro-inflammatory chemokines TNFα, IL-8, and MCP1 were significantly decreased with MegaDuo™ treatment (versus M-SHIME^®^ control); no other significant differences were observed. We speculate that the decrease in TNFα, IL-8, and MCP1 were the result of positive effects of the MegaDuo™ probiotics on the abundance of Firmicutes and Bacteroidetes, as both are known to produce SCFAs [43,44], which have an anti-inflammatory effect [12].

It seems necessary for dysbiosis of the normal gut microbiome to occur for a probiotic to exert observable benefits in a relatively short (2 week) treatment period. One of the most common side-effects of antibiotic treatment is antibiotic associated diarrhea, which is thought to be caused by a drastic change in the intestinal microbiome (i.e., dysbiosis) [45]. In a clinical trial, *B. subtilis* was shown to reduce the incidence and severity of antibiotic associated diarrhea in patients receiving antibiotic treatment [46]. However, there is a lack of knowledge regarding the mechanism(s) responsible for this improvement. The results from the present study help bridge this gap by providing mechanistic insight. We observed a reduction in TNFα and MCP1 along with protection from LPS-induced barrier damage in an in vitro model of antibiotic-induced gut microbiome dysbiosis after treatment with the probiotic MegaDuo™. These results suggest that treatment with the probiotic MegaDuo™ may reduce cytokine and chemokine production and provide protection from barrier damage under conditions of antibiotic-induced dysbiosis. While these results are of interest, additional studies using fecal microbiota from multiple healthy donors are necessary to further elucidate and validate these findings.

Gut barrier integrity is critically important for healthy intestinal function. One protective mechanism of a healthy gut barrier is the separation of the luminal contents from the underlying immune cells by mucus and a single layer of epithelial cells. The integrity of this membrane is crucial to prevent infection as well as unnecessary, uncontrolled intestinal inflammation. Many gut-associated disorders are characterized by inflammation and a compromised gut barrier [47]. Given the reduction in TNFα and improvement in gut barrier function after probiotic treatment observed in our study, we think that studies of the effects of MegaDuo™ in gut-associated disorders are warranted. A previous clinical trial reported that *B. coagulans* GBI-30, 6086 probiotic treatment decreased abdominal pain and bloating in IBD patients [48], providing some support for the beneficial effects of probiotic treatment in gut-associated disorders.

Patients having other diseases associated with compromised gut barrier integrity have experienced improvement with *Bacillus* spp. probiotic use, again with little understanding of the mechanisms behind this improvement. Probiotic treatment with *B. coagulans* GBI-30, 6086 resulted in quality of life improvement and reduction of gastrointestinal symptoms in a clinical trial of patients with post-prandial gas-related symptoms [49]. Another clinical trial reported a reduction of daily bowel movements in patients with IBD who were treated with *B. coagulans* GBI-30, 6086 [50]. Our study provides some much-needed insight into possible mechanisms behind the clinical improvements observed with *Bacillus* spp. probiotic treatment and provides support for testing the effects of MegaDuo™, a unique combination of *B. coagulans* SC208 and *B. subtilis* HU58, in patients with gut-associated disorders, particularly in cases where the normal gut microbiome is no longer intact.

Limitations of our study include the fact that the results were obtained using the gut microbiome of a single healthy donor and that the only stressor studied was antibiotic-induced dysbiosis. While M-SHIME^®^ experiments with the aim of generalizing the effect of a treatment in a given population frequently include multiple donors to account for the potential effects of interindividual variability, the present study aimed to evaluate the impact of a test product when comparing the same donor under normal conditions and conditions of antibiotic-induced dysbiosis. Therefore, we think that the data presented are self-standing, representing an exploratory data set that can be used to guide larger, confirmatory studies. Additionally, M-SHIME^®^ control suspensions from Week 1 were suspected to have been contaminated during sample processing (i.e., after sample collection). Because of this, we used data from Week 2 as a comparator for Week 1. While we acknowledge that this could have influenced the interpretation of the results, we expect this to be minimal, as the microbiota composition for the M-SHIME^®^ control was similar between Weeks 1 and 2 (Figure 2 and Figure 3, TR1 vs. TR2).

Overall, this study showed that under conditions of antibiotic-induced dysbiosis in the M-SHIME^®^ system, suspensions from MegaDuo™ treated colonic chambers could significantly reduce gut membrane barrier damage and significantly decrease TNFα, MCP1, and IL-6 compared with the M-SHIME^®^ control (no probiotic treatment) in an in vitro co-culture system representing the gut mucosal barrier. The greatest effect was observed when the M-SHIME^®^ suspensions were collected after 2 weeks of treatment with MegaDuo^®^.

## Figures and Tables

**Table 1:** Effect of M-SHIME^®^ supernatants on cytokine and chemokine release in the co-culture system under conditions of dysbiosis.

Cytokine/Chemokine	Dysbiosis (Clindamycin Pre-treatment)					
	Week 1			Week 2		
	M-SHIME^®^ Control	MegaDuo™ Treated	*p* Value	M-SHIME^®^ Control	MegaDuo™ Treated	*p* Value
TNFα ^a^	6585.5 (194.7)	6970.0 (305.4)	0.8227	9172.6 (622.3)	6604.7 (343.9)	0.0067
IL-8 ^b^	336.2 (50.2)	343.7 (51.2)	0.9906	372.3 (21.5)	391.9 (29.6)	0.9502
MCP1 ^a^	7068.4 (1129.6)	4071.1 (696.0)	0.0699	8388.8 (601.0)	8032.9 (835.3)	0.9480
CXCL10 ^a^	781.5 (78.6)	641.5 (124.3)	0.4049	563.8 (29.4)	555.21 (23.5)	0.9962
IL-10 ^a^	10.3 (0.4)	10.7 (0.9)	0.8938	9.9 (0.8)	12.1 (0.4)	0.0777
IL-6 ^a^	510.6 (14.7)	370.3 (26.4)	0.0070	483.5 (24.0)	483.9 (29.5)	>0.999

All values are given as mean (standard error of the mean). ^a^ Values are in pg/mL. ^b^ Values are in ng/mL. M-SHIME^®^ = mucosal simulator of the human intestinal microbial ecosystem.

**Table 2:** Effect of M-SHIME^®^ supernatants on cytokine and chemokine release in the co-culture system under healthy conditions.

Cytokine/Chemokine	Healthy					
	Week 1			Week 2		
	M-SHIME^®^ Control	MegaDuo™ Treated	*p* Value ^1^	M-SHIME^®^ Control	MegaDuo™ Treated	*p* Value
TNFα ^2^	n.d.	5827.8 (202.2)	0.0047	7251.4 (275.6)	7124.6 (137.4)	0.9140
IL-8 ^3^	n.d.	249.6 (61.6)	0.0238	427.3 (22.0)	372.9 (35.8)	0.5785
MCP1 ^2^	n.d.	5778.6 (331.5)	0.0359	8595.7 (339.3)	11,516.0 (1212.1)	0.0304
CXCL10 ^2^	n.d.	645.3 (23.8)	0.9326	655.9 (22.5)	703.0 (18.5)	0.3057
IL-10 ^2^	n.d.	14.9 (1.3)	0.3786	17.0 (1.0)	19.8 (1.0)	0.2049
IL-6 ^2^	n.d.	746.4 (34.4)	0.9165	764.7 (31.9)	865.7 (36.5)	0.1294

All values are given as mean (standard error of the mean). ^1^ Week 1 MegaDuo™ treated vs. Week 2 M-SHIME^®^ control; Week 1 M-SHIME^®^ control suspensions were suspected to have been contaminated during sample processing (i.e., after sample collection) so the data could not be used for analysis. The microbiota composition was similar for the control M-SHIME vessel at Week 1 and Week 2 (Figure 2 and Figure 3), allowing for the use of Week 2 data as a comparator in place of Week 1 data. ^2^ Values are in pg/mL. ^3^ Values are in ng/mL. M-SHIME^®^ = mucosal simulator of the human intestinal microbial ecosystem.

## Supplementary Materials

The following are available online at https://www.mdpi.com/2076-2607/8/7/1028/s1, Table S1: Reciprocal Simpson Diversity Index in the lumen and mucus of the proximal colon (M-SHIME®) treated with MegaDuo™, Figure S1: Barrier integrity of Caco-2 cells (controls), Figure S2: Effect of controls on cytokine and chemokine levels in the Caco-2/THP1-Blue™ co-culture model, Figure S3: Effect of controls on NFκB activity of PMA-treated THP1 cells after LPS stimulation in the Caco-2/THP1-Blue™ co-culture model.
