# Supplementary material for: Bacillus subtilis HU58 and Bacillus coagulans SC208 Probiotics Reduced the Effects of Antibiotic-Induced Gut Microbiome Dysbiosis in an M-SHIME® Model
Source: Microorganisms. 2020 Jul 11;8(7):1028. doi: 10.3390/microorganisms8071028 (PMC7409217; doi:10.3390/microorganisms8071028)
Supplement: Supplementary file 1 [file microorganisms-08-01028-s001.pdf]

microorganisms

Supplementary Material

1 Supplementary Table

Supplementary Table 1. Reciprocal Simpson Diversity Index in the lumen and mucus of the proximal colon (M-SHIME®) treated with MegaDuo™

	Healthy		Antibiotic-induced dysbiosis		
	C3	TR2	C2	AB	TR2
Luminal microbial community	4.3	3.9	4.8	3.9	4.0
Mucosal microbial community	3.5	4.5	8.0	3.6	6.4

C2 = control Week 2; C3/AB = control Week 3 (healthy condition, pre-treatment)/antibiotic (antibiotic-induced dysbiosis condition, pre-treatment); TR1 = treatment Week 1; TR2 = treatment Week 2; M-SHIME® = mucosal simulator of the human intestinal microbial ecosystem.

2 Supplementary Figures

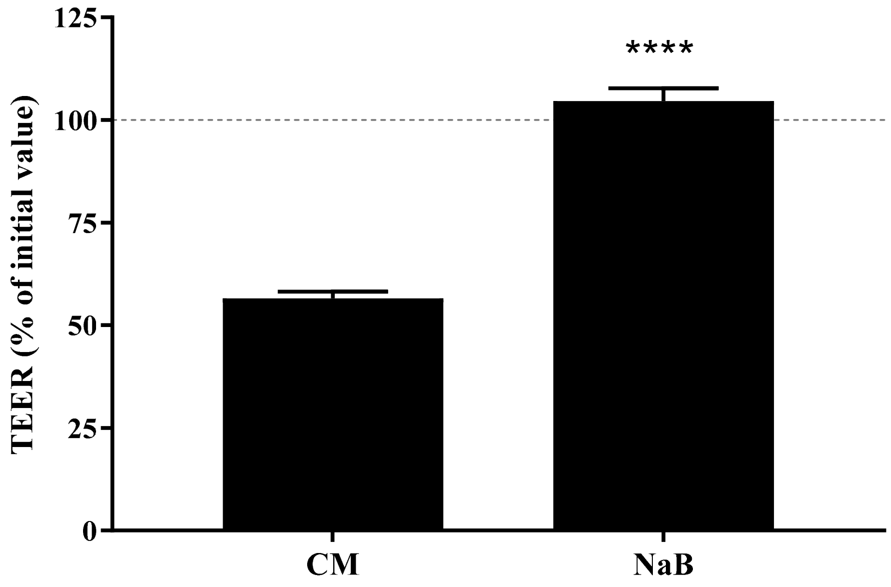

Supplementary Figure 1. Barrier integrity of Caco-2 cells (controls)

Caco-2/THP1-Blue™ co-cultures were set up by placing an insert with a monolayer of Caco-2 cells into a well of PMA-differentiated (100 ng/mL, 48 h) THP1-Blue™ cells which were then exposed to CM or 12 mM NaB. After 24 h, THP1-induced damage to the Caco-2 cells was measured and reported as percent of initial TEER value. Error bars represent SEM (n = 3); complete Caco-2 media (negative) and NaB (positive) values were compared using an unpaired, two-tailed Student's t-test. $^{****}p \leq 0.0001$, gray dashed line = 100% TEER; Abbreviations: CM, Caco-2 media; PMA, phorbol 12-myristate 13-acetic acid; NaB, sodium butyrate; SEM, standard error of the mean; TEER, transepithelial electric resistance

A

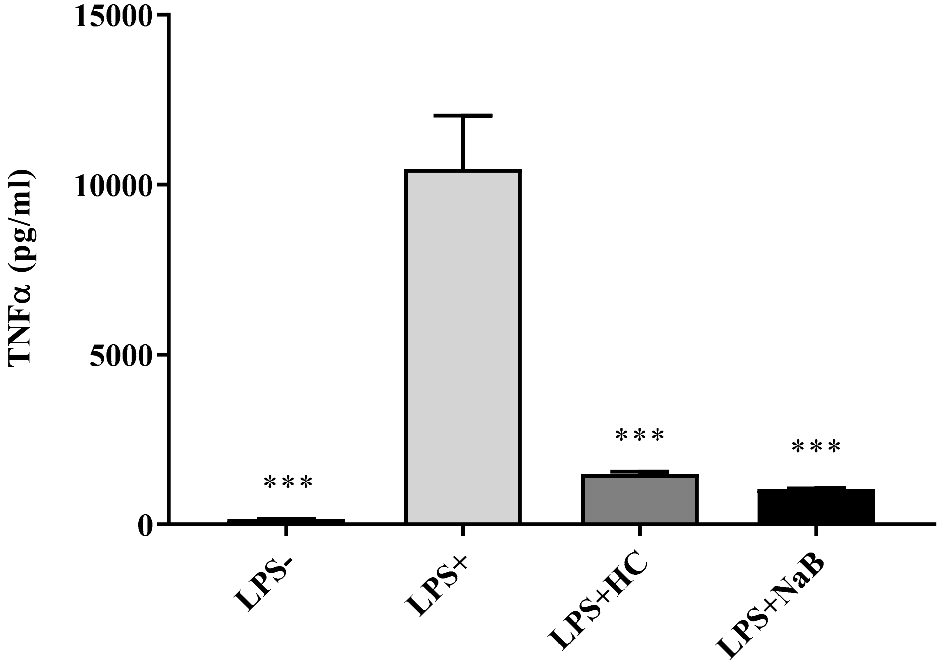
15000
10000
5000
0
TNFα (pg/ml)
***
***
***
LPS-
LPS+
LPS+HC
LPS+NaB

B

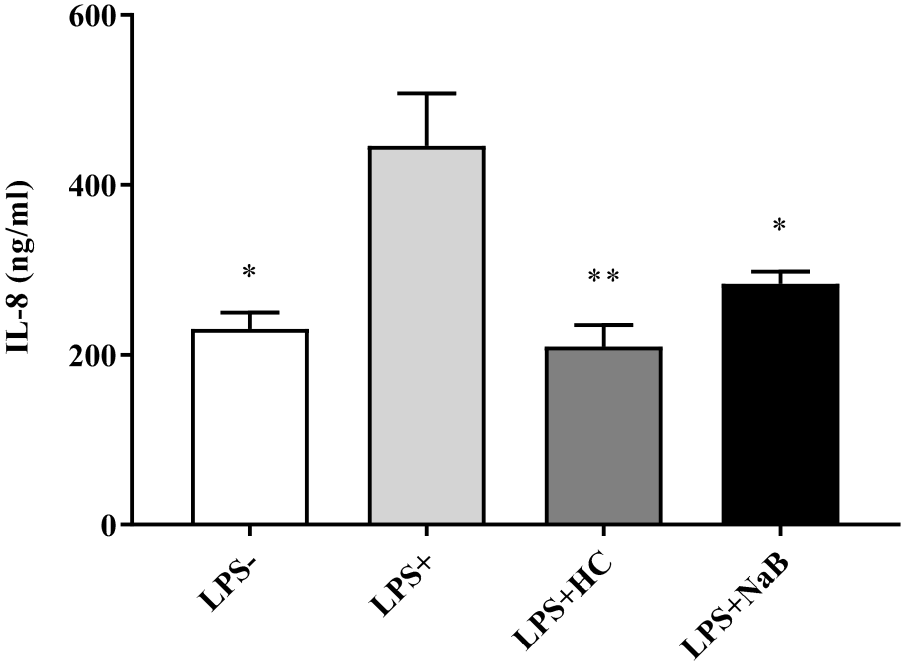
600
400
200
0
IL-8 (ng/ml)
*
**
*
LPS-
LPS+
LPS+HC
LPS+NaB

C

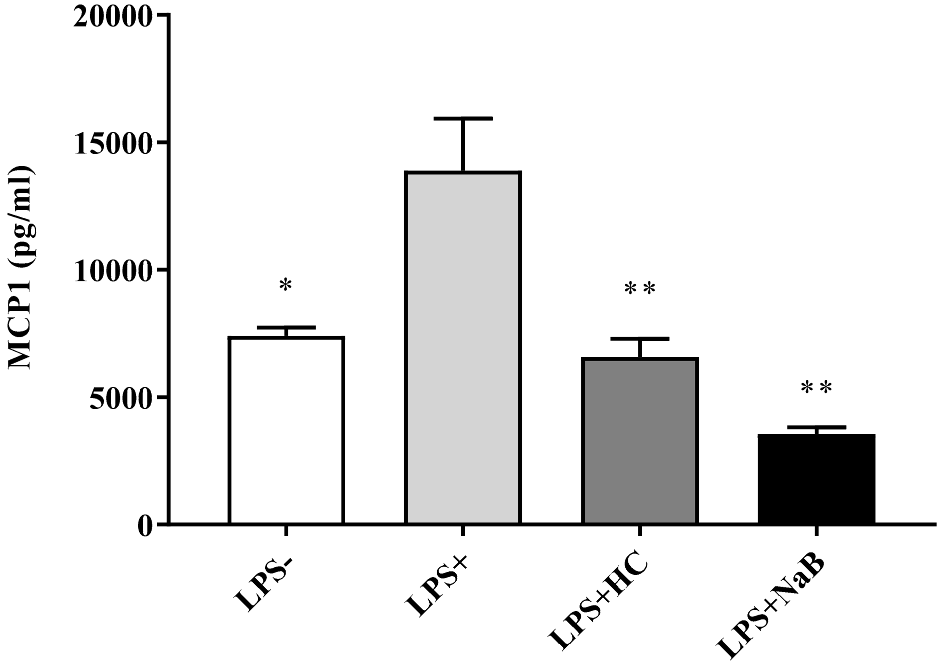
20000
15000
10000
5000
0
MCP1 (pg/ml)
*
**
**
LPS-
LPS+
LPS+HC
LPS+NaB

D

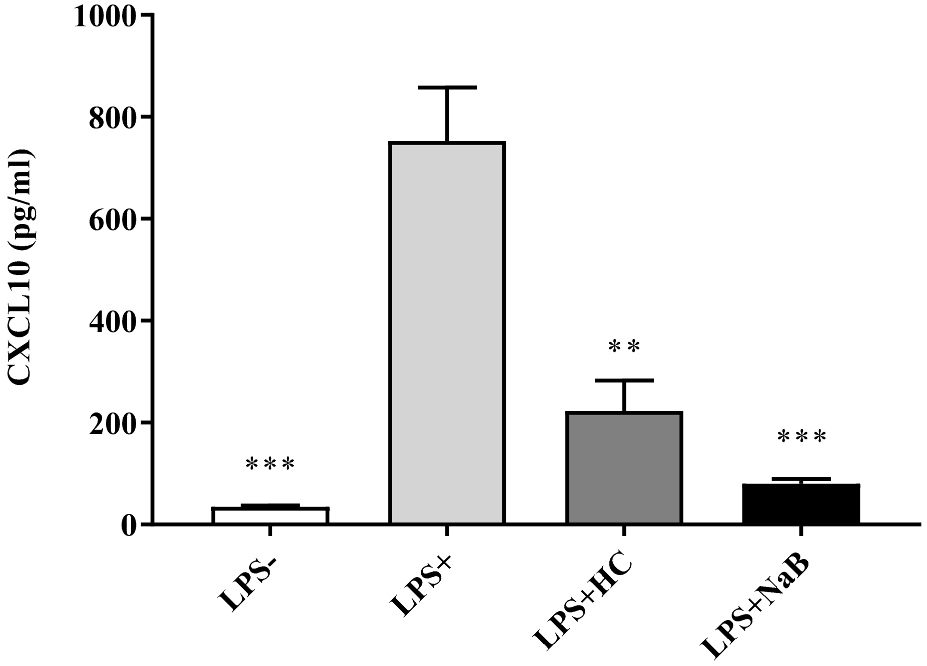
1000
800
600
400
200
0
CXCL10 (pg/ml)
***
**
***
LPS-
LPS+
LPS+HC
LPS+NaB

E

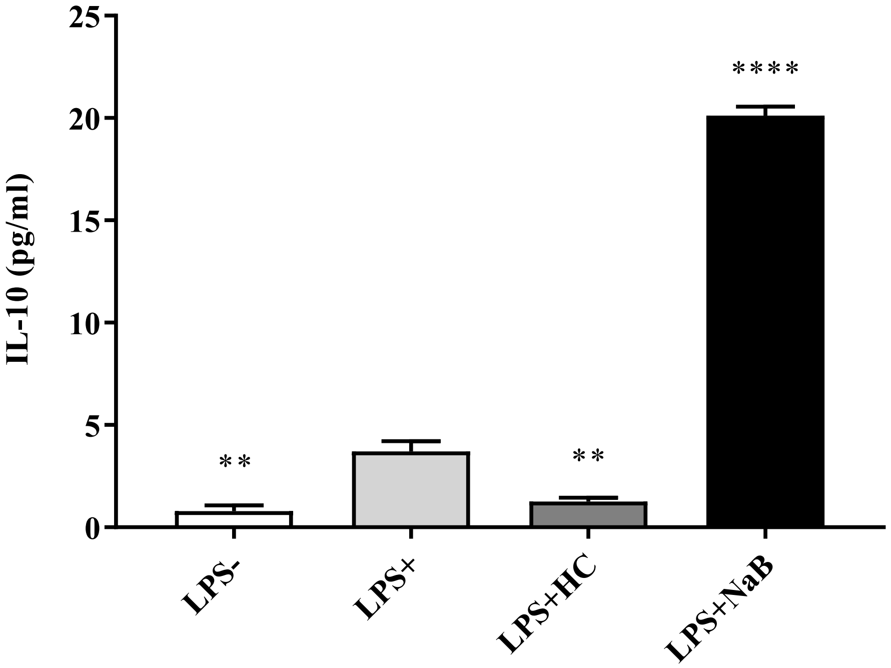

IL-10 (pg/ml)
25
20
15
10
5
0
**
****
**
****
LPS-
LPS+
LPS+HC
LPS+NaB

F

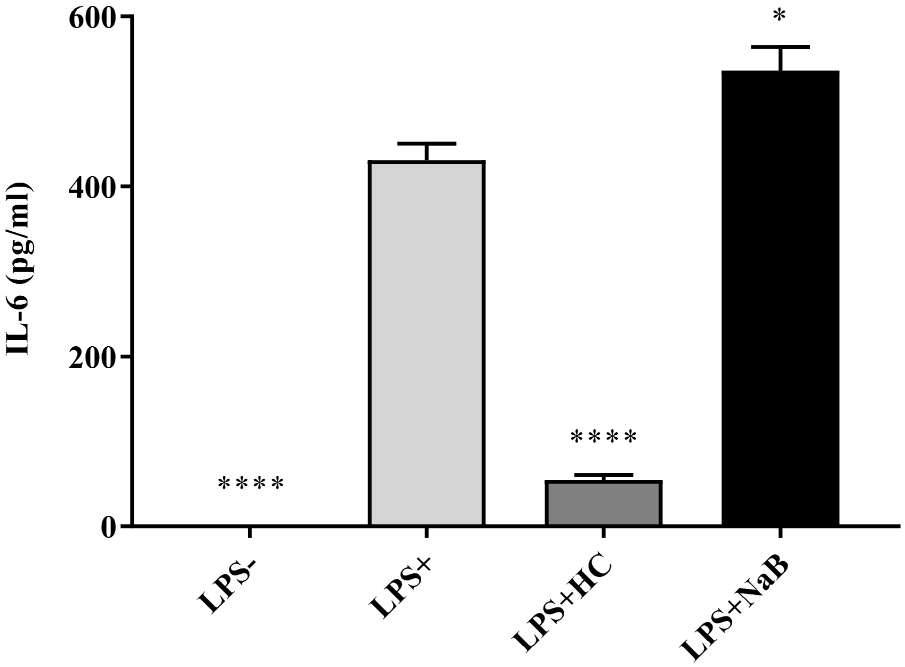

IL-6 (pg/ml)
600
400
200
0
****
****
*
LPS-
LPS+
LPS+HC
LPS+NaB

Supplementary Figure 2. Effect of controls on cytokine and chemokine levels in the Caco-2/THP1-Blue™ co-culture model

Caco-2/THP1-Blue™ co-cultures were set up by placing an insert with a monolayer of Caco-2 cells into a well of PMA-differentiated (100 ng/mL, 48 h) THP1-Blue™ cells which were then exposed to CM. After 24 h, the basolateral media was discarded and replaced with CM containing 500 ng/mL ultrapure LPS stimulate the cells. For controls, some wells received LPS and 1 µM HC, LPS and 12 mM NaB, or CM (no LPS). After 6 h, basolateral supernatants were collected, and chemokine/cytokine levels were measured. (**A**) TNFα, (**B**) IL-8, (**C**) MCP-1, (**D**) CXCL10, (**E**) IL-10, and (**F**) IL-6. Error bars represent SEM (n = 3); LPS-, LPS+HC, LPS+NaB were compared to LPS+ using an ordinary one-way ANOVA with Dunnett's multiple comparisons test. $^{*}p \leq 0.05$, $^{**}p \leq 0.01$, $^{***}p \leq 0.001$; Abbreviations: CM, Caco-2 media; HC, hydrocortisone; LPS, lipopolysaccharide; NaB, sodium butyrate; SEM, standard error of the mean

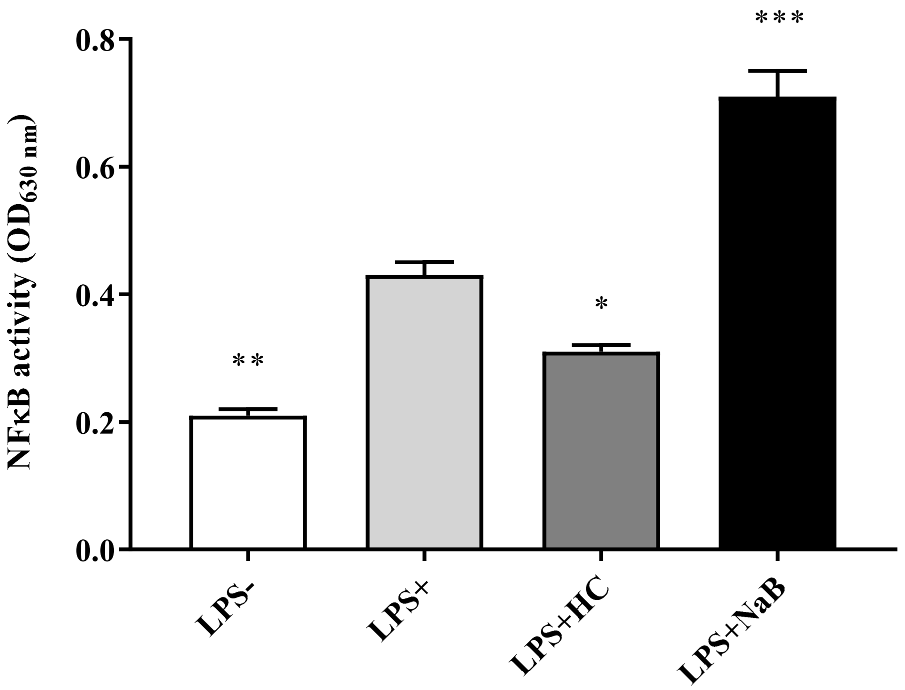

Supplementary Figure 3. Effect of controls on NFκB activity of PMA-treated THP1 cells after LPS stimulation in the Caco-2/THP1-Blue™ co-culture model

Caco-2/THP1-Blue™ co-cultures were set up by placing an insert with a monolayer of Caco-2 cells into a well of PMA-differentiated (100 ng/mL, 48 h) THP1-Blue™ cells which were then exposed to CM. After 24 h, the basolateral media was discarded and replaced with CM containing 500 ng/mL ultrapure LPS stimulate the cells. For controls, some wells received LPS and 1 μM HC, LPS and 12 mM NaB, or CM. After 6 h, basolateral supernatants were collected and NFκB activity was measured. Error bars represent SEM (n = 3); LPS-, LPS+HC, LPS+NaB were compared to LPS+ using an ordinary one-way ANOVA with Dunnett's multiple comparisons test. $^{***}p \leq 0.001$; Abbreviations: CM, Caco-2 media; HC, hydrocortisone; LPS, lipopolysaccharide; NaB, sodium butyrate; SEM, standard error of the mean
